# Mechanomyogram for Muscle Function Assessment: A Review

**DOI:** 10.1371/journal.pone.0058902

**Published:** 2013-03-11

**Authors:** Md. Anamul Islam, Kenneth Sundaraj, R. Badlishah Ahmad, Nizam Uddin Ahamed

**Affiliations:** AI-Rehab Research Group, Universiti Malaysia Perlis (UniMAP), Kompleks Pauh Putra, Arau, Perlis, Malaysia; University of Sydney, Australia

## Abstract

**Background:**

Mechanomyography (MMG) has been extensively applied in clinical and experimental practice to examine muscle characteristics including muscle function (MF), prosthesis and/or switch control, signal processing, physiological exercise, and medical rehabilitation. Despite several existing MMG studies of MF, there has not yet been a review of these. This study aimed to determine the current status on the use of MMG in measuring the conditions of MFs.

**Methodology/Principal Findings:**

Five electronic databases were extensively searched for potentially eligible studies published between 2003 and 2012. Two authors independently assessed selected articles using an MS-Word based form created for this review. Several domains (name of muscle, study type, sensor type, subject's types, muscle contraction, measured parameters, frequency range, hardware and software, signal processing and statistical analysis, results, applications, authors' conclusions and recommendations for future work) were extracted for further analysis. From a total of 2184 citations 119 were selected for full-text evaluation and 36 studies of MFs were identified. The systematic results find sufficient evidence that MMG may be used for assessing muscle fatigue, strength, and balance. This review also provides reason to believe that MMG may be used to examine muscle actions during movements and for monitoring muscle activities under various types of exercise paradigms.

**Conclusions/Significance:**

Overall judging from the increasing number of articles in recent years, this review reports sufficient evidence that MMG is increasingly being used in different aspects of MF. Thus, MMG may be applied as a useful tool to examine diverse conditions of muscle activity. However, the existing studies which examined MMG for MFs were confined to a small sample size of healthy population. Therefore, future work is needed to investigate MMG, in examining MFs between a sufficient number of healthy subjects and neuromuscular patients.

## Introduction

Researchers are exploring to set suitable methods to examine muscles' activities noninvasively; these methods for example, include surface electromyogram (sEMG) [1], [2], sonomyogram (SMG) [3], [4], tensiomyogram (TMG) [5], [6], and mechanomyogram (MMG) [7], [8].

Although the widely used sEMG has attracted attention for decades as a reliable tool for the assessment of skeletal muscles, it has some drawbacks. sEMG is sensitive to external noise and interference, which limits its operating environment and range of application [9]. In addition, sEMG sensor requires low noise and a very stable signal component [9]. Furthermore, signal processing and analysis is complex [9], [10]. It is also expensive, since it requires three electrodes for differential recordings [9].

Conversely, MMG has been proposed as another tool to study muscle mechanical activity [11]. The term mechanomyography represents a technique by which the mechanical activity of muscle is detected using specific transducers to record muscle surface oscillations due to mechanical activity of the motor units [12]. MMG signals can be detected using several types of transducers including piezoelectric contact sensors (PIZ) [13]–[15], microphones (MIC) [7], [16], [17], accelerometers (ACC) [18]–[22] and laser distance sensors (LDS) [23]–[25].

MMG can provide some notable advantages over sEMG [26]. First, due to its propagating property through the muscle tissue, the placement of MMG sensors is not required to be precise or specific [27]. Second, MMG is a mechanical signal; thus, it is not influenced by the change in the skin impedance due to sweating [9], [26]. Further, MMG can be used in conjunction with sEMG to examine neuromuscular function [28].

However, with the aid of MMG, many researchers have continued to explore its application aspects. There are many examples where MMG has been applied to characterize muscle activity. These applications include for example, the characterization of MFs [22], [29]–[31], the development of prosthesis and/or switch control [32], [33], studying activity of motor unit [34]–[36], evaluating muscles during sports and exercises [37]–[39], monitoring neuromuscular blockade [40], and development of a suitable model for studying the motor unit activity [41]. In addition, we have found several studies which conducted research on MMG sensor development [6], [15], [42], simultaneously testing multiple sensors and comparing their effects on muscles' [43], [44]. We also have found several studies that evaluated the sensor placement effect on muscles [19], [45]. Several works on MMG signal processing for muscle characterization [16], [46], [47] have already been reported.

However, we only found three MMG related review articles [28], [48], [49], which examined MMG amplitude and frequency responses [49], as well as motor unit recruitment strategies [28] and motor unit firing rates [48] during dynamic muscle actions. To our knowledge, there has not been an article which presents a review of MMG for monitoring MFs. This gap motivates the present study. Therefore, the purpose of this review was to determine the current status on the use of MMG in assessing MF. Another objective was to use this review outcome to identify priority area(s) for future research.

## Methods

### Searching

We performed a comprehensive literature search for MMG of MF assessment in the Elsevier, PubMed, IEEE, SpringerLink, and Google Scholar electronic databases for relevant articles published from 2003 to 2012. We deliberately set our search strategy (the details of the search strategy may be obtained from Table S1) to be broad in the combined search of: # 1 (mechanomyography) AND # 2 (systematic review AND review) AND # 3 (muscle-function) AND # 4 (muscle-assessment) NOT # 5 (electromyography). There were no language and category restrictions during databases searching. Journal articles, conferences, books, letters, and clinical reports were examined for potentially eligible studies. In addition, we checked the reference lists of all important articles that were retrieved in the search.

### Study selection

The title and abstract of studies identified by the search were screened for potential relevance. The full text of all potentially relevant studies was reviewed to determine if they fulfilled the eligible criteria. We included studies that described a theoretical or practical of only MMG for MF assessment. Two authors (MAI and NUA) independently screened the results of the electronic searches to select potentially relevant citations based on the title and abstract according to the criteria defined above. The studies those meet the most relevant criteria best were included for this review. The studies those were written in a language other than English, and articles those examined animal muscles were eliminated. In addition, we excluded articles that lacked in-depth discussions, without proper data presentation, and with unclear or vague descriptions of the protocol used.

### Data extraction

Two authors (MAI and KS) abstracted data individually using an MS-Word structured data extraction form specially created for this review (Table S2). The extracted data were compared and discussed by the two authors before being compiled as final information. Information extracted from each article included: i) name of the studied muscle and its contraction type, ii) sensor type and model, iii) MFs and subject types, iv) measured parameters, algorithms and hardware, v) main results, vi) author comments, vi) recommended applications and vii) priority for future research.

### Validity assessment

Three authors (MAI, KS and BA) analyzed the data extracted from the potentially relevant articles. After analysis we decided to depend on information extracted from the most relevant studies; those that were organized with proper data presentation, clearly verified selection of protocols, and through demonstration of research methodologies, to reduce risk of bias.

### Quantitative data synthesis

There was a significant number of MMG driven MF assessment studies across areas of muscle fatigue, strength, muscle balance, muscle movement activities, and effects on muscle conditions for practicing exercises. We described these studies qualitatively and present detailed results in tabulated form (Table 1–Table 7).

**Table 1:** An overview of MMG driven fatigue test from the biceps brachii muscle.

Study	Sensors	Subjects	Parameters	Assessment	Results
Tanaka et al. (2011)	PIZ	2 healthy males	MPF and variance of MMG	Fatigue	Rate of increase of variance with time declined and peak of MPF with time reached quickly for fatigue subject.
Comment: i) Insufficient sample size and lack of details; ii) The development MMG sensor enables monitoring of muscle condition like fatigue via evaluation parameters as MPF and variance.					
Future work: Not suggested.					
Xie et al. (2010)	ACC	5 healthy human subjects	Embedded (m) and correlation dimension (D_2_)	Fatigue	D_2_ increased with m initially and then entered into a flat area at slight fluctuation.
Comment: i) Insufficient sample size and lack of details; ii) MMG is a high-dimensional chaotic signal which supports the use of nonlinear dynamics theory for analysis and modelling of fatigue.					
Future work: Combining the surrogate data method with chaotic invariants may be potentially applied to differentiate the muscle states.					
Hendrix et al. (2010)	ACC	10 adults (4 males and 6 females, mean age: 22.0±2.1 years)	MPF of MMG and sEMG, and critical torque (CT)	Fatigue threshold	There were no significant differences between fatigue thresholds (CT, sEMG MPF_FT_ and MMG MPF_FT_), and the mean torque values (Nm) from the three fatigue thresholds were significantly inter-correlated at r = 0.94–0.96.
Comment: i) Small sample size and unclear whether subjects are healthy or not; ii) MMG and sEMG MPF may be useful to examine fatigue threshold noninvasively.					
Future work: Future studies should examine sEMG and MMG MPF responses during continuous muscle actions at sEMG MPF_FT_ and MMG MPF_FT_ to directly validate these tests.					
Xie et al. (2009)	ACC	5 subjects	m and D2	Fatigue signal nature	MMG signals in fatigue state of all observed subjects were a chaotic signal, and were generated by nonlinear dynamics systems.
Comment: i) Insufficient sample size and lack of details; ii) MMG is a high-dimensional chaotic signal which supports the use of nonlinear dynamics theory for the analysis and modelling MMG signals.					
Future work: Not suggested.					
Feng et al. (2009)	MIC	5 healthy subjects, (age: from 21 to 32 years; 4 males and 1 female)	%MVC, RMS and MNF of MMG	Fatigue and muscle activity	RMS increased with increase in the force of contraction. There was significant change in the RMS and consistent decrease in the value of MMG with the onset of fatigue.
Comment: i) Insufficient sample size of healthy subjects and imbalance with sex; ii) There is a consistent decrease in the RMS value of MMG with the increase of muscle fatigue but the MNF of the MMG is observed to be very inconsistent and hence not a useful tool to measure muscle fatigue.					
Future work: Need to improve the understanding of the size and location of MIC, and determine the impact of gel applied to the surface of the MIC prior to determining the efficacy of MMG to identify muscle activity.					
Krizaj et al. (2008)	LDS	13 healthy males (age: from 19 to 42 years)	Muscle belly displacement, sustain and half relaxation times	Fatigue rate	For all parameters, ICC were above 0.86 which meant good short-term repeatability and normalized standard error was lower than 2% which meant high precision.
Comment: i) Small sample size and only healthy subjects; ii) Maximal displacement and half relaxation time show largest influence on muscle fatigue rate and hence are expected to be the best measure of fatigue rate.					
Future work: Further studies of long-term repeatability should be performed.					
Madeleine et al. (2006)	ACC and MIC	14 healthy males (right-handed; age: 26.7±4.9 years)	RMS, MPF, power spectral variance (Mc2), and skewness (µ3)	Fatigue	For both sensors, absolute and normalized RMS and Mc2 increased with contraction time while MPF and µ3 decreased. The rates of change of RMS over time were significantly correlated for both but not correlated for spectral moments.
Comment: i) Small sample size and only healthy subjects; ii) Higher order spectral moments of the MMG signal change during sustained contraction, indicating a complex modification in the shape of the power spectrum.					
Future work: Not suggested.					
Beck et al. (2005)	PIZ	7 males (age: 23±3 years)	Repetition number, MPF, MDF and centre frequency (CF) of MMG and sEMG	Fatigue	Significant correlation between MPF, MDF and CF for both sEMG and MMG. All these parameters decreased with increase in repetition number.
Comment: i) Insufficient sample size and unclear whether subjects are healthy or not; ii) Fourier based methods are acceptable for determining the patterns for normalized MMG and sEMG CF during fatiguing dynamic muscle actions.					
Future work: Not suggested.					
Gregori et al. (2003)	sEMG and PIZ coupled	Normal subjects	MMG and sEMG amplitude	Sensor development for fatigue	Differential amplification significantly improved the signal-to-noise ratio in MMG recordings and significantly suppressed artifacts.
Comment: i) Unclear sample size and lack of details; ii) The new composite probe records muscular activity more efficiently than non-differential probes.					
Future work: Not suggested.					

**Table 7:** Overview of MMG measurement in exercises.

Study	Sensors	Muscles	Subjects	Parameters	Assessment	Results
Esposito et al. (2011)	ACC	MG	11 healthy males (age: 22±1 years)	RMS and MNF of MMG and sEMG	Stretching effects on MMG	After stretching, no significant change was found by sEMG, MMGp–p and slope decreased (16% and 10%, respectively) and remained depressed for the entire recovery period. MMG RMS increased (20%) and returned to pre-stretching values within 15 minutes.
Comment: i) Small sample size and only healthy subjects; ii) Stretching significantly alters MMG and force signals. Also, MMG RMS returns to pre-stretching values which suggests that changes in viscoelastic parallel components recover after a few minutes.Future work: Further investigations aimed at elucidating the role of transverse muscle tendon unit stiffness on MMG amplitude are required.						
Malek et al. (2011)	ACC	VM	10 healthy, males (age: 24.4±1.3 years)	MPF and amplitude of MMG and output power	CE exercise effects on muscle's IZ	MMG amplitude was not influenced by IZ but MMG MPF changed depending on distal, IZ and proximal sensors on the muscle for each subject.
Comment: i) Small sample size and only healthy subjects; ii) IZ does not influence the MMG signal from VM muscle during CE.Future work: VM may be used in future studies of muscular fatigue without regard for signal contamination by the IZ.						
Herda et al. (2010)	ACC	VL	5 RT (age: 23±3 years) 5 AT (age: 32±5 years) and 5 SED (age: 23±4 years); healthy males	RMS of MMG and sEMG, and force	Fiber type discrimination among three trainings	AT group had the highest percentage of type I fiber area, the RT group had the highest percentage of type IIa fiber area, and the SED group had the highest percentage of type IIx fiber area.
Comment: i) Small sample size and only healthy subjects; ii) The present findings suggest that the information provided by both the MMG RMS and sEMG RMS vs. force relationships is unique. The log-transformed MMG RMS vs. force relationship may offer an attractive, noninvasive model for statically examining changes in the motor unit activation strategies.Future work: Not suggested.						
Malek et al. (2010)	ACC and PIZ	VL and RF	9 healthy, college-aged males (age: 23.6±0.8 years)	Output power, MMG amplitude and MMG MPF	CE exercise effects on MMG sensors	CE exercise influenced similar effect on MMG amplitude but was inconsistent for MMG MPF for both sensors and muscle groups.
Comment: i) Small sample size and only healthy subjects; ii) MMG amplitude responses for both muscles for incremental CE are the same when comparing PIZ and ACC sensors on a subject-by-subject basis.Future work: Not suggested.						
Taylor et al. (2010)	ACC	Thigh and shin	9 healthy subjects (4 males and 5 females, varying in height and weight)	Sample vs. acceleration	Monitoring correct exercise for knee osteoarthritis	The results obtained a reliable average accuracy (0.92, 0.97, and 0.90 respectively) and an acceptable average sensitivity (above 70%) of the standing hamstring curl, reverse hip abduction, and lying straight leg raise when performing within subject and across subjects cross validation.
Comment: i) Small sample size and only healthy subjects; ii) The system will provide feedback on exercise performance based on the classifier decisions, motivate the patient to continue exercise, and report patient progress back to a physician and/or care giver.Future work: Need to examine this experiment with patients who are currently undergoing physical therapy to evaluate the results with healthy subjects.						
Malek et al. (2009)	ACC	RF	8 healthy males (age: 27.3±2.3 years)	Output power, and MMG amplitude and MPF	Knee extensor (KE) and CE muscle action	KE resulted in similar patterns of responses with CE for MMG amplitude of the composite data in all 8 subjects, but MPF was inconsistent.
Comment: i) Small sample size and only healthy subjects; ii) KE, rather than the traditional CE exercise may be an optimal mode of examining MMG amplitude for the RF muscle.Future work: Future studies are needed to examine the motor control strategies of the quadriceps muscles for dynamic exercises and these should use the KE model and report the normalized MMG amplitude data only, whereas the CE model should be used to examine neuromuscular fatigue during cycling.						
McKay et al. (2007)	ACC	RF	10 healthy, moderately fit young males (age: 23.0±2.3 years)	RMS of MMG and sEMG, and normalized MMG amplitude over time	Exercise effect on muscle mechanical signal	Importantly, all subjects demonstrated an increase of MMG signal ranging from 1.8 to 7.7 times of the pre-exercise level.
Comment: i) Small sample size and only healthy subjects; ii) Resting muscle is more mechanically active following resistance exercise and this may contribute to an elevated oxygen consumption.Future work: Need to examine whether resting-muscle MMG changes with muscle disease or with alterations in muscle tone or atrophy.						
Cramer et al. (2007)	ACC	RF	10 females (age: 23.0±2.9 years) and 8 males (age: 21.4±3.0 years)	MMG and sEMG amplitudes, joint angle and pT	Stretching effect on muscle strength	pT, acceleration time, and sEMG amplitude decreased from pre to post-stretching at 1.04 and 5.23 rad/s. There was no change in work, joint angle at pT, isokinetic range of motion, or MMG amplitude.
Comment: i) Small sample size and only healthy subjects; ii) Static stretching appears to affect muscle strength at slow and fast speeds, and thus may affect all types of athletes.Future work: Need to examine the volume of stretching necessary to safely increase joint range of motion before performance, but not elicit detrimental changes in muscle force production that could adversely affect performance.						
McKay et al. (2006)	ACC	RF	10 fairly healthy subjects (6 males and 4 females; age: 33±13 years)	MMG and work	Exercise activity	MMG and work was linearly correlated, non-exercised thigh showed half of MMG activity compare to exercised thigh, MMG activity was higher at shorter length of RF muscle.
Comment: i) Small sample size and only healthy subjects; ii) The greater MMG activity at shorter muscle lengths suggests that muscle that is less stretched could more freely oscillate, producing higher MMG amplitudes.Future work: Further evidence is needed to examine that excess post-exercise resting MMG activity is likely neurally mediated.						

## Results

### Flow of included study

The comprehensive literature search returned a total of 36 articles (Figure 1). Of these, 16 met the inclusion criteria as muscle fatigue, three met as strength, one met the inclusion criteria as muscle balance, whereas seven met as muscle movement activities, and the remaining nine met the inclusion criteria as muscle exercise or stretching.

**Figure:**
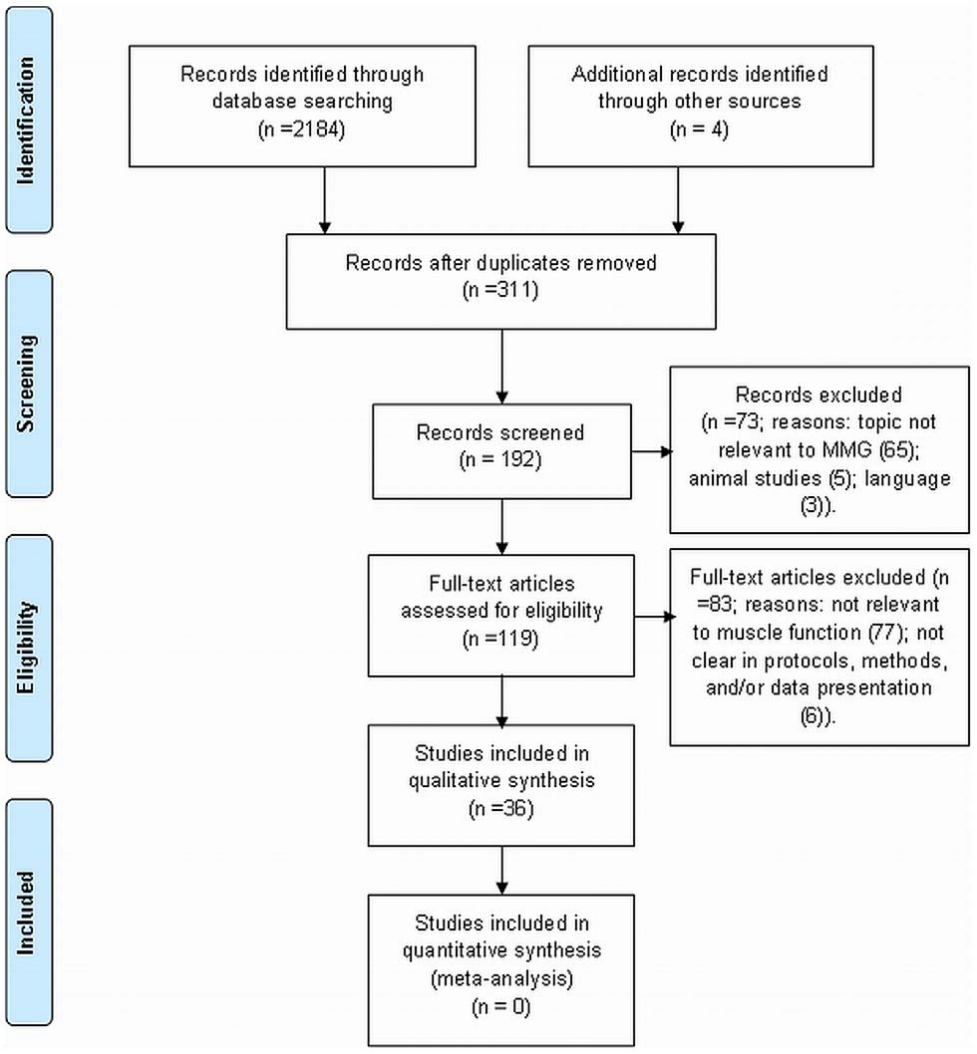
Flow chart of the process of selecting potential studies.

### Study characteristics

Among the 36 relevant studies, 30 studies were peer-reviewed journal articles, six studies were conference papers. Nine [13], [15], [21], [42], [50]–[54] involved fatigue assessment from upper arm (biceps brachii, BB) muscles, five studies [14], [29], [30], [55], [56] were related to fatigue assessment of leg muscles (quadriceps), one [57] conducted fatigue investigation of the masseter muscle and another [58] presented fatigue measurements of both BB and vastus lateralis (VL) muscles. Three studies [59]–[61] incorporated strength tests from VL, rectus femoris (RF), and BB muscles. Another study [31] included muscle balance investigation of quadriceps muscles. Seven studies [7], [22], [33], [62]–[65] involved muscle activities of movements from different muscles, and the rest nine [8], [37]–[39], [44], [66]–[69] were related to investigation of MFs of diverse exercises from quadriceps muscles.

### Fatigue assessment

Table 1 presents the main details of fatigue measurement of nine relevant studies using MMG from BB muscle. Gregori et al. [42] developed a new surface composite probe for differential MMG and sEMG recording in order to measure muscle fatigue from BB. A similar non-differential MMG sensor was used for comparisons. The new sensor recorded muscular activity more efficiently than the non-differential probe and could therefore be useful in studying fatigue and neuromuscular diseases [42]. Tanaka et al. [15] developed a PIZ to monitor muscle fatigue for the biceps and triceps brachii muscles. The authors reported that they were able to measure muscle fatigue using the developed sensor [15].

Beck et al. [13] performed a comparison between the fast Fourier transform and the discrete wavelet transform for determining MMG and sEMG centre frequency patterns during fatiguing isokinetic muscle actions of the BB muscle. The authors suggested that Fourier-based methods were acceptable for determining the patterns for normalized MMG and sEMG centre frequency during fatiguing dynamic muscle actions at moderate velocities [13].

Madeleine et al. [54] analyzed the trends of the first three power spectral moments of the MMG signals recorded from the BB muscle using a MIC and an ACC during sustained fatiguing contractions at 30% of maximum voluntary contractions (MVC). For both the MIC and ACC, absolute and normalized root mean square (RMS) values and power spectral variance increased while mean frequency (MNF) and skewness decreased with contraction time [54]. Feng et al. [51] reported an experimental study of MIC-based MMG signal intensity against force of contraction and muscle fatigue during cyclic contractions of the BB muscle. They observed that MMG signal intensity decreased with the increase of onset of muscle fatigue [51]. Furthermore, Krizaj et al. [53] claimed maximum displacement and half relaxation time as parameters to measure muscle fatigue for the BB muscle.

Xie et al. [21] explored fatigue MMG signals during static contractions by applying nonlinear dynamic analysis methods for the BB muscle. The results advocated the use of nonlinear dynamics theory (Volterra–Wiener–Korenberg model and noise titration approach) for analysis and modelling of fatiguing MMG [21]. Again, Xie et al. [52] investigated linear and non-linear properties of MMG signal detected from the BB muscle during fatiguing isometric contractions. They reported that MMG signal during fatigue was nonlinear from all subjects [52].

Hendrix et al. [50] examined fatigue threshold from the BB muscle by comparing the critical torque threshold, electromyographic mean power frequency fatigue threshold (sEMG MPF_FT_), and mechanomyographic mean power frequency fatigue threshold (MMG MPF_FT_). They suggested that there were no significant differences between these fatigue thresholds, and the mean torque values from the three fatigue thresholds were significantly intercorrelated at r = 0.94–0.96. Thus, activated motor units may be examined by using non-invasive methods like the MMG MPF_FT_ test [50].

However, five relevant studies focused on fatigue assessment from the quadriceps muscles (Table 2). Ebersole and Malek [14] examined the relationship between fatigue and electromechanical efficiency (EME) for the VL and vastus medialis (VM) muscles and found 58% and 66% decreases in EME for the VM and VL respectively, concurrently with a 47% decline in torque production. They concluded that in healthy muscles, the EME of both muscles decreased concurrently with a decrease in torque [14].

**Table 2:** An overview of fatiguing MMG assessment for quadriceps muscles.

Study	Sensors	Subjects	Parameters	Assessment	Results
Armstrong (2011)	ACC	10 healthy subjects (gender balanced, age: 25±3 years)	Intensity, wavelet index and frequency	Fatigue and postural control	Peak MMG intensity was at lower frequency 12 Hz for male, and valley intensity was at higher frequency 42 Hz for female. Intensity increased with fatigue.
Comment: i) Small sample size and only healthy subjects; ii) MMG intensity analysis is useful for posture control and studying fatigue from the VL, VM and RF muscles. Future work: The relationship between Piper-rhythm, changes in constraints affecting postural control, and changes in MMG (and sEMG) intensity need to be warranted.					
Hendrix et al. (2010)	ACC	9 adults (4 males and 5 females; age: 21.6±1.2 years)	MMG MPF_FT_ and torque	Fatigue threshold	There were no differences in the isometric torque levels associated with the MMG MPF_FT_ for the three superficial (VL, VM, and RF) muscles of the quadriceps.
Comment: i) Small sample size of partially athletic subjects; ii) The MMG MPF_FT_ test may provide a non-invasive method to examine fatigue of quadriceps muscles during isometric muscle actions. Future work: Future studies should compare the effects of continuous isometric, intermittent isometric and dynamic muscle actions on differences in the MMG MPF_FT_ of the VL, VM, and RF muscles.					
Al-Zahrani et al. (2009)	ACC	31 healthy subjects (15 males; age: 32.3±7.6 years, 16 females; age: 30.3±10.3 years)	RMS, MPF, MDF and ICC	Reliability of fatigue test within day and between days	Low reliability and large error for between days of MPF and MDF respectively. Overall, ICC was highly reliable for MPF with lower SDD for MDF.
Comment: i) Sample size with details is reliable but only healthy subjects; ii) MMG RMS, MPF and MDF linear regression slopes from the RF muscle are not suitable for monitoring muscle fatigue between days on healthy subjects due to the SDD values.					
Future work: Not suggested.					
Faller et al. (2009)	ACC	10 healthy males (age: 26.7±5.35 years)	RMS, MPF of MMG and torque	Fatigue at the presence of NMES	At present NMES, MMG RMS correlated with torque but MMG MPF did not significantly correlate with torque.
Comment: i) Small sample size and only healthy subjects; ii) MMG for fatigue assessment of RF muscle during application of NMES protocol can be simultaneously applied due to absence of electrical interference and it can be used during functional movements in the NMES-generated muscle contractions. Future work: Not suggested.					
Ebersole et al. (2008)	PIZ	10 healthy males (age: 23.2±1.2 years)	Torque and EME	Fatigue	Linear regression confirmed the decrease in torque and EME for VM and VL. EME slopes were same for VM and VL.
Comment: i) Small sample size and only healthy subjects; ii) EME may offer insight into the influence on fatigue of skeletal muscle function and be a useful tool to assess and quantify clinically relevant asymmetries in VM and VL muscles functions.Future work: Future research is needed to examine EME for these muscles in a clinical population as well as in response to specific interventions.					

Faller et al. [55] studied using triaxial-based ACC for MMG signal from the RF muscle to assess fatigue during the execution of neuromuscular electrical stimulation (NMES) protocol, which is used widely for rehabilitation in the physical therapy of fatigue caused by excessive voluntary contraction. They confirmed that the RMS value of MMG correlated with torque but mean power frequency (MPF) of MMG did not significantly correlate for the present NMES protocol [55].

Al-Zahrani et al. [56] investigated the reliability in assessing RF muscle fatigue within-day and between-days using triaxial ACC-assisted MMG. They found poor reliability [56] in between-days for fatigue assessment. The poor between-days reliability found in this study suggests caution in using MMG RMS, MPF, median frequency (MDF) and their corresponding regression slopes in assessing muscle fatigue due to the high number of smallest detectable difference (SDD) values [56].

Hendrix et al. [30] tested MMG MPF_FT_ from the VL, VM and RF muscles during each fatiguing isometric muscle action. They determined that there were no significant differences among the MMG MPF_FT_ values for the three muscles. Hence, the MMG MPF_FT_ test may provide a non-invasive method to examine the fatiguing effects during isometric muscle actions [30].

Armstrong [29] studied the intensity analysis of Morlet wavelets of MMG signal as an alternative to power spectral analysis for the evaluation of postural control strategy during the single-legged stance and to examine the effects of fatigue over the VL, soleus and VM muscles. He found that the intensity of MMG signals increased with increasing fatigue [29]. Furthermore, he mentioned that intensity analysis is a useful tool for exploring posture control and fatigue study [29].

One study by Gobbo et al. [58] verified twitching torque and MMG peak-to-peak (MMGp-p) amplitude from both dominant BB and VL muscles by inducing fatiguing stimulation to investigate muscle fatigue. Another relevant study by Ioi et al. [57] showed supporting evidence of using EME as a parameter to measure masseter muscle fatigue. Table 3 presents an overview of the two relevant studies of fatigue assessment.

**Table 3:** An overview of MMG-driven fatigue test.

Study	Sensors	Muscles	Subjects	Parameters	Assessment	Results
Ioi et al. (2006)	Amorphous with small magnet	Masseter	16 healthy Japanese males (age: 25.6±2.3 years)	Bite-force, EME and average rectified value (ARV)	Fatigue during bite force	ARV for MMG raised up to 20% and then started to fall. A nonlinear and linear relationship between MVC and ARV of pre and post fatigue for MMG and sEMG respectively was observed. EME was lower at post fatigue.
Comment: i) Small sample size and only healthy subjects; ii) MMG analysis combined with sEMG may be a more useful method for evaluating the masseter muscle fatigue.						
Future work: The relationship between force and the MMG activity is to be warranted.						
Gobbo et al. (2006)	ACC	BB and VL	10 healthy sedentary males (age: from 20 to 50 years)	Peak torque (pT) and MMGp-p	Fatigue during electrical stimulation (ES)	MMGp-p and %pT decreased more in the VL muscle with increasing fatigue. %pT and MMGp-p had a high correlation for both the BB and VL muscles.
Comment: i) Small sample size and confined to healthy sedentary males only; ii) For both muscles MMGp-p and pT decreased with increasing fatigue. It may be useful in practical applications for monitoring mechanical fatigue growth, in order to avoid potential stress disorders.						
Future work: Not suggested.						

### Strength assessment

Three studies examined muscle strength [59]–[61] (Table 4). Matta et al. [60] addressed the temporal (RMS) and spectral components of MMG signals from the BB muscle of males and females during different levels of contraction to characterize muscle strength. Another study by Marek et al. [61] focused on examining the short-term effects of static and proprioceptive neuromuscular facilitation stretching on muscle strength and output power. They concluded that both static and proprioceptive neuromuscular facilitation stretching caused similar deficits in strength, power output, and muscle activation at both slow (60°/s) and fast (300°/s) velocities [61]. In addition, Ryan et al. [59] examined the inter-individual variability for the patterns of responses of MMG amplitude and MPF versus isometric torque from the VL muscle in lower-strength and higher-strength individuals. The authors indicated that the composite MMG amplitude versus isometric torque relationship was best fit with a linear model for the lower-strength group and a cubic model for the higher-strength group [59]. They also found that the composite MMG MPF versus isometric torque relationships was best fit with linear models for both the groups [59].

**Table 4:** An overview of muscle strength assessment.

Study	Sensors	Muscles	Subjects	Parameters	Assessment	Results
Ryan et al. (2007)	ACC	VL	12 healthy males (age: 25±4 years)	MPF, RMS of MMG and torque	Muscle strength	MMG amplitude versus isometric torque relationship was best fit with a linear model for the lower strength group and a cubic model for the higher strength group. MMG MPF was best fit with a linear model for both the groups.
Comment: i) Small sample size and only healthy subjects; ii) Differences in strength do not affect the patterns of responses for MMG amplitude or MPF.						
Future work: Future studies should examine the individual patterns of response to draw conclusions about motor control strategies.						
Marek et al. (2005)	ACC	VL and RF	10 females (age: 23±3 years) and 9 males (age: 21±3 years); apparently healthy subjects	MMG amplitude, pT and mean power (MP)	Strength at slow and fast speeds	MMG amplitude increased for the RF muscle at 60°/s static stretching but remained unchanged in all other cases.
Comment: i) Relatively small sample size and only healthy subjects; ii) Both static and proprioceptive neuromuscular facilitated stretching caused similar deficits in strength, power output, and muscle activation at both slow (60°/s) and fast (300°/s) velocities.Future work: Further research is needed to examine the effects of pre-exercise stretching on muscle strengthening and/or strength assessments in athletes or patients who have experienced a muscle, tendon, or joint injury.						
Matta et al. (2005)	ACC	BB	15 males (age: 24.0±5.25 years) and 12 females (age: 21.7±1.5 years); healthy subjects	RMS and MNF of MMG signal	Strength of male and female	RMS in X-axis and Y-axis increased with workload for both male and female, but MNF for male was almost stable and slightly decreased for female with workload in both the axes.
Comment: i) Relatively small sample size and only healthy subjects; ii) RMS in X-axis and Y-axis of the ACC sensor increased with workload for both male and female, but MNF for male was almost stable and slightly decreased for female with workload for both the axes.						
Future work: Not suggested.						

### Muscle balance assessment

One study included muscle balance measurement (Table 5). Armstrong et al. [31] evaluated the reliability of a protocol for using a microelectromechanical high-resolution ACC to measure centre of mass accelerations in the three cardinal planes (vertical, medial/lateral and anterior/posterior) and uniaxial ACC to measure MMG for the purpose of assessing balance and postural control. High resolution ACC and MMG offered reliable information pertaining to balance, and may have application in evaluating postural control and stability [31].

**Table 5:** Overview of MMG for assessing muscle balance.

Study	Sensors	Muscles	Subjects	Parameters	Assessment	Results
Armstrong et al. (2010)	ACC	VL, VM and soleus	5 males and 5 females (mean age: 25±3 years); healthy subjects	MMGp-p and ICC	Balance and postural control	Almost all measurements demonstrated moderate-to-strong reliability in examining balance.
Comment: i) Small sample size and only healthy subjects; ii) MMG provides reliable information pertaining to balance, and may have application in evaluating postural control and stability.						
Future work: Need to determine relationships and predictability of these measures in a controlled quasi-static positioning with more dynamic motions and fatigue states.						

### Muscle movement assessment

Seven studies [7], [22], [33], [62]–[65] examined muscle activities due to movements as shown in Table 6. Scheeren et al. [64] investigated the functional movement of RF and VL muscles using MMG between healthy and spinal cord injured (SCI) patients during different functional electrical stimulation (FES) profiles. The authors found that the MMG signal was different between healthy and SCI patients but comparable in the RF and VL muscles per subject [64]. In addition, Krueger et al. [62] made a correlation between MMG signal and passive movements of healthy and SCI patients. The correlation found by the authors was positive for healthy subjects and negative for SCI patients [62]. Tian et al. [63] also observed different sEMG and MMG behaviours accompanied with age-related sarcopenia for elder and younger group collected from the VL muscle during concentric contraction with movement intensities of 45%, 60%, and 75%. The averages MMG RMS between groups were different for all movement intensities while sEMG RMS was indistinguishable between groups. sEMG MNF and MMG MNF increased with movement intensity among both the young and the elderly subjects [63]. The authors suggested that MMG should be used as an important tool in studying muscle contraction in age-related sarcopenia [63]. Yoshimi et al. [65] developed a new system to examine muscle activities and mandibular movement patterns during sleep bruxism (tapping, clenching and grinding). The system consisted of a dual-axis ACC and sEMG to record activities of the masseter muscle. The authors showed that grinding (59.5%) was most common, followed by clenching (35.6%) based on relative activity to MVC, whereas tapping was only 4.9%. They concluded that tapping, clenching, and grinding movements of the mandible could be effectively differentiated by the new system and sleep bruxism was predominantly perceived as clenching and grinding which varied between individuals [65]. Kawakami et al. [7] further investigated MMG and sEMG signals in the human lateral pterygoid muscle during mandibular movements for maximum voluntary clenching. They showed that the activity of the lateral pterygoid muscle could be evaluated by MMG signals recorded in the external ear canal if the jaw closing major muscles do not show active contractions [7]. Furthermore, Alves and Chau [33] designed and tested MMG signals during eyebrow movements to control a binary switch. They showed that the eyebrow movement MMG-driven switch performed with almost perfect sensitivity and specificity for all participants. The performance of their algorithm was robust against typical participant movements [33]. However, Scheeren et al. [22] characterized wrist movements like elbow extension and flexion, ulnar deviation and radial deviation. Their statistical analysis indicated that flexion was different from extension, ulnar and radial deviation, and radial deviation was different from ulnar deviation and flexion [22].

**Table 6:** Overview of MMG for muscle movement activities assessment.

Study	Sensors	Muscles	Subjects	Parameters	Assessment	Results
Kawakami et al. (2012)	MIC	Lateral pterygoid	3 healthy males(age: 29.3±2.5 years)	sEMG amplitude and MMG amplitude	Movement activity during clenching	MMG and sEMG amplitudes correlated for both 20 mm and 30 mm jaw movements but not for 10 mm and maximal clenching lateral pterygoid muscle movements.
Comment: i) Insufficient sample size and only healthy subjects; ii) The activity of the lateral pterygoid muscle may not be evaluated using MMG, especially during the jaw movements under strong contraction of the jaw closing muscles.Future work: Not suggested.						
Krueger et al. (2011)	ACC	RF and VL	12 healthy subjects (age: 31.45±4.56 years) and 13 SCI patients (age: 32.06±9.46 years)	RMS, MNF and skewness of MMG, and knee angle	Knee angular movement	The correlation between MMG MNF and MMG RMS in healthy subjects was classified as positive, and it was classified as weak in SCI patients.
Comment: i) Both neuromuscular patients and healthy subjects are compared; ii) MMG skewness and MMG MNF are spectral analysis features, and they showed antagonist responses to knee angle during passive movements.Future work: Not suggested.						
Alves and Cahu, (2010)	MIC and ACC coupled	Frontalis	10 healthy subjects (5 males; age: 27±2 years)	Time vs. RMS value of MMG, and frequency vs. CWT for 4 eyebrow movements	Movement activities to control binary switch	The average sensitivity and specificity of the MMG-driven switch was 99.7±0.4% and 99.9±0.1%, respectively.
Comment: i) Small sample size and only healthy subjects; ii) The frontalis muscle is a suitable site for controlling the MMG-driven switch during eyebrow movement.Future work: Further investigation of the potential benefits of MMG-driven control for the target population is warranted.						
Scheeren et al. (2010)	ACC	RF and VL	10 healthy males (age: 28.3±6.6 years) and 3 SCI male patients (age: 34.4±9.8 years)	RMS and MNF of MMG	Muscle functional movement	The lowest values for MMG RMS and MNF parameters were verified in the 200–50 FES profile suggesting less muscle modification during the experiment.
Comment: i) Small sample size and unbalanced number of neuromuscular patients and healthy subjects; ii) This study may be helpful in creating experimental setups with FES walking performances and control strategies of artificial functional movements.Future work: Not suggested.						
Tian et al. (2010)	ACC	VL	10 healthy elderly (age: 64.0±4.5 years) and 10 healthy young adults (age: 22.0±2.8 years)	RMS and MNF of both MMG and sEMG, and movement intensity	Age-related sarcopenia	The MMG RMS showed differences between the young and the elderly across all three intensity levels whereas sEMG RMS differed only at the greatest intensity.
Comment: i) Small sample size and only healthy subjects; ii) Although all four main parameters, sEMG RMS, MMG RMS, sEMG MNF and MMG MNF, show different results for diverse movement intensities and group demographics, MMG is still a more sensitive measurement tool to examine age-related sarcopenia.Future work: Not suggested.						
Scheeren et al. (2010)	ACC	Forearm muscles	20 healthy males (age: 24.0±5.5 years)	MMG RMS, peak counting and zero crossing	Wrist movement	MMG signals of flexion differed from extension, ulnar and radial deviations, and radial deviation differed from ulnar deviation and flexion.
Comment: i) Small sample size and only healthy subjects; ii) The ability to identify distinct wrist movements using two or more MMG sensors brings good perspective to the development of new control strategy algorithms for driving upper-limb prostheses.Future work: Need to study larger number of limb movements for control strategy, such as pronation and supination of the forearm, or discrimination of fine movements or each finger individually.						
Yoshimi et al. (2009)	ACC	Masseter	19 healthy subjects (16 males and 3 females, age: 28.5±5.8 years)	Amplitude of MMG and sEMG, muscle activity vs. bruxism length	Movement due to sleep bruxism	Tapping was a rhythmic muscle activity with Y-axis movement, clenching was a strong muscle activity with no Y-axis movement, and grinding was a muscle activity with X-axis and Y-axis movements.
Comment: i) Small sample size and imbalance in gender types; ii) Tapping, clenching, and grinding movements of the mandible could be effectively differentiated by the new system.Future work: Not suggested.						

### MMG measurement in exercises

Nine studies [8], [37]–[39], [44], [66]–[69] reported MMG measurements of exercise. Table 7 depicts an overview of MMG effects examined for exercises. McKay et al. [39] performed recording of MMG signals to determine the effects of graded levels of exercise on ipsilateral and contralateral post-exercise resting RF muscle. They observed that MMG activity was greater when the RF muscle length was shorter (i.e. when the leg was extended versus flexed). This result suggested that less stretched muscles could more freely oscillate, producing higher MMG amplitudes [39]. Again, McKay et al. [69] examined MMG signals from resting muscles before and after resistance exercises. They found that resting MMG amplitudes increased about threefold after vigorous resistance exercise, and that the increase decayed exponentially over time. Conversely, resting muscle sEMG amplitudes doubled after resistance exercises, but their amplitudes were below the resolution of the measuring instrument [69]. Further, Herda et al. [67] utilized MMG signal to discriminate muscle fiber types during three training phases namely, resistance trained (RT), aerobically trained (AT), and sedentary (SED). The authors showed that there were differences in fiber type composition of the VL muscle among AT, RT, and SED persons [67].

However, Cramer et al. [38] investigated the acute effects of static stretching on neuromuscular functions (peak torque, work, joint angle at peak torque, acceleration time, range of motion, sEMG amplitude, and MMG amplitude) during maximal concentric isokinetic leg extensions in men and women. After stretching, the authors found that peak torque, acceleration time, and sEMG amplitude decreased from pre-stretching to post-stretching at 1.04 and 5.23 rad/s; there were no changes in work, joint angle at peak torque, isokinetic range of motion, or MMG amplitude [38]. Moreover, Esposito et al. [37] evaluated the effects of stretching of the MG muscle on sEMG signal, MMG signal, and muscle force. They found that stretching may affect the mechanical properties of the muscle but no significant change was found in case of sEMG [37]. Another work by Taylor et al. [66] described an ACC-based system that can detect and classify small deviation from a correct exercise performance for knee osteoarthritis (joint disorder caused by pain and stiffness). This system allowed the possibility of quick recovery and prevention for the patients by taking full advantage of exercise secondary disorders [66].

Malek et al. [68] examined the MMG amplitude and MPF versus power output relationships for the RF muscle during cycle ergometry (CE) and knee extensor (KE) incremental exercises on the same subject. They demonstrated that the KE model expressed similar patterns of responses (best-fit with linear model) for absolute and normalized MMG amplitude of the composite data in all eight subjects, whereas for the CE exercise, these patterns varied on a subject-to-subject basis. In the analysis of MMG MPF, there were no consistent patterns of responses for CE and KE exercises involving the RF muscle [68]. Further, Malek et al. [44] compared ACC and PIZ MMG sensors for VL and RF muscles during incremental CE. They showed similar patterns of response for MMG amplitude but inconsistent responses for MMG MPF by both the sensors and muscle groups on a subjects-to-subject basis [44]. Malek et al. [8] however examined the effect on MMG responses during incremental CE across innervation zone (IZ) of the VM muscle. They concluded in their findings that MMG signals during dynamic exercise were not influenced by IZ of the VM muscle [8].

## Discussion

This review summarizes the findings of MMG in measuring diverse MFs. The main findings of this review are as follows: First, we find sufficient studies that MMG may be used to measure different conditions of MFs including muscle fatigue, strength, and balance. Second, this review provides sufficient evidence that MMG may be used to examine muscle actions due to movements, even for patients with SCI. Third, we also find sufficient studies to show that MMG is able to monitor muscle activities under various types of exercise paradigms. Fourth, this review reveals an important issue that all of these studies, except one [56] (Table 1 to Table 7), have been conducted on small samples of healthy subjects only. For medical diagnostic purposes, comparisons would be needed with well-defined samples of neuromuscular patients. Finally, since we included studies that had a proper data presentation, clearly verified protocols and thoroughly demonstrated research methodologies, we believe that there is a little risk of bias across studies.

### Fatigue or strength assessment

As mentioned previously, MMG provides a platform for muscle fatigue, and strength assessment. In most cases, researchers consider MMG amplitude, MPF, torque and contraction force to measure fatigue [15], [51], [54], [55], and strength [59]–[61]. However, Al-Zahrani et al. [56] left caution of using RMS, MPF, and MDF for fatigue measurement between days due to the low number of smallest detectable difference. They used intraclass correlation coefficients (ICC) instead [56]. Xie et al. [21] explored fatigue MMG signals using embedded and correlation dimension parameters and advocated the use of nonlinear dynamics theory (Volterra–Wiener–Korenberg model and noise titration approach). Armstrong [29] performed intensity analysis using Morlet wavelets of MMG signal to determine postural control and fatigue. However, Krizaj et al. [53] claimed maximum displacement and half relaxation time as parameters to measure muscle fatigue. On the other hand, the authors showed supporting evidence for using EME as a parameter to measure muscle fatigue [14], [57]. The choice of parameters used for muscle fatigue measurement differ possibly due to the various set of protocols considered, use of different MMG sensors or selection of different muscles. Nevertheless, all of these studies agreed that MMG may be implemented as a measurement tool for monitoring muscle fatigue. However, all but Al-Zahrani et al. [56] examined muscle fatigue using a small sample size which were confined to healthy subjects only.

### Movement activity or balance assessment

Based on the studies reviewed, MMG appears to be an effective means of measuring muscle balance, and activities due to movement. Researchers in [7], [62]–[65] allowed MMG RMS amplitude and MNF as the parameters to examine muscle activities of movements. However, Scheeren et al. [22] analyzed zero crossing, peak counting and RMS of MMG signals to characterize wrist movements. The results from their studies suggest that MMG may be capable of measuring muscle movement activities caused by either SCI or age-related sarcopenia, and muscle balance of quadriceps. In addition, MMG may also be useful to differentiate mandible movements during sleep [65], and voluntary clenching tasks [7]. Alves and Chau [33] also detected eyebrow movements using MMG with high accuracy. All of these studies confirm that MMG may be used to examine movement activities of different muscles. On the other hand, Armstrong et al. [31] conceded that ICC besides the commonly used MMGp-p amplitude, could be chosen to characterize muscle balance. However, all these assessments used a small sample size of healthy subjects.

### MMG in exercise assessment

We have found studies which include MMG measurement to examine muscle activities of exercises. This review reveals that stretching may affect the response of MMG signals. The authors in [38], [39], for example, clearly showed the effects on MMG amplitude for the RF muscle after stretching. Further, Esposito et al. [37] found that stretching may affect the mechanical properties of the MG muscle. However, Malek et al. [68] demonstrated that there is a linear response between MMG and knee extensor exercises for all participated eight subjects, but the MMG response was influenced on a subject-to-subject basis for CE exercises involving the RF muscle. Again, Malek et al. [44] determined that MMG amplitude responded in similar patterns using PIZ and ACC sensors from VL and RF muscles on subject-to-subject basis. This subject dependent MMG response during CE may be due to varying muscle fiber composition, and different force labels production between subjects. Further, McKay et al. [69] demonstrated that the resting RF muscle affected MMG signals before and after resistance exercises. Furthermore, Taylor et al. [66] implemented and studied the effect of exercises on MMG to monitor the correct amount of exercise required for knee osteoarthritis subjects. However, Herda et al. [67] analyzed the effects on MMG RMS of the VL muscle during AT, RT, and SED and used these to identify muscle fiber types resulting from the three training phases. On the other hand, Malek and Coburn [8] found no influence on MMG response during incremental CE exercises across the IZ of the VM muscle. This result suggests that MMG signal from the VM muscle during CE exercises may be used to study muscle condition without regards to signal contamination by the IZ. Thus, this review reports that MMG may be applied as a useful tool to monitor muscle activities for stretching or exercising. However, all of these studies only conducted the assessments using a small sample size of healthy population.

### Limitation

This review comprises several strong points, including its uniqueness. To date, this is the first study specially designed to retrieve, analyze and critically appraise existing trends of MFs assessment based on MMG. There are nevertheless some limitations in this study. We included studies which still suffer from limited sample size, poor characterization of subjects and the heterogeneous methodology. We also did not include non-English studies in our analysis. On the other hand, lower quality of trials not published in English may also introduce bias [70]. In addition, a small amount of publication bias could also be present due to the bounded time frame (2003–2012) used for article searching. This bias can be neglected because technology was much less advanced in earlier years and can hardly be compared to approaches used in later studies. Overall, the information documented by this article may be useful to future researchers in understanding the existing status on the use of MMG for monitoring MFs.

## Conclusions

In summary this review reveals that MMG may be applied as a useful tool to examine muscle fatigue, strength, and balance. We also find sufficient evidence that MMG may be used to evaluate muscle activities of movements. In addition, this review also shows that MMG may be a useful tool to monitor muscle activities during, after or before exercises. However, we observe that of these 36 studies, only two studies performed analyses using neuromuscular patients but still with a small sample size, and only one examined with a reliable sample size that is also confined to a healthy population only. Therefore, future work is needed to examine MMG for MFs in a sufficient number between healthy subjects and neuromuscular patients.

## Supporting Information

Table S1 **Keywords and search strategies for MMG in measuring muscle function.**
(DOC)

Table S2 **Data extraction form for MMG in assessing muscle function.**
(DOC)
