# Supplementary material for: Mechanomyogram for Muscle Function Assessment: A Review
Source: PLoS One. 2013 Mar 11;8(3):e58902. doi: 10.1371/journal.pone.0058902 (PMC3594217; doi:10.1371/journal.pone.0058902)
Supplement: Table S1 — Keywords and search strategies for MMG in measuring muscle function. (DOC) [file pone.0058902.s001.doc]

**Table S1.** Keywords and search strategies for MMG in measuring muscle function.

| **Databases** | **Trials** | **Limitations** | | **Citations returned** | **Total citations returned** | **Final most relevant articles** |
| --- | --- | --- | --- | --- | --- | --- |
|  |  | **Year** | **Category** |  |  |  |
| IEEE | Step 1 | 2003-2012 | All fields | 73 | 2184 | 36 |
|  | Step 2 |  |  | 2 |  |  |
|  | Step 3 |  |  | 56 |  |  |
|  | Step 4 |  |  | 33 |  |  |
|  | Step 5 |  |  | 4 |  |  |
| Elsevier | Step 1 |  |  | 247 |  |  |
|  | Step 2 |  |  | 48 |  |  |
|  | Step 3 |  |  | 215 |  |  |
|  | Step 4 |  |  | 171 |  |  |
|  | Step 5 |  |  | 92 |  |  |
| SpringerLink | Step 1 |  | All categories | 202 |  |  |
|  | Step2 |  |  | 28 |  |  |
|  | Step 3 |  |  | 138 |  |  |
|  | Step 4 |  |  | 0 |  |  |
|  | Step 5 |  |  | 98 |  |  |
| PubMed | Step 1 |  | All fields | 208 |  |  |
|  | Step 2 |  |  | 10 |  |  |
|  | Step 3 |  |  | 146 |  |  |
|  | Step 4 |  |  | 12 |  |  |
|  | Step 5 |  |  | 4 |  |  |
| Google scholar | Step 1 |  |  | 1450 |  |  |
|  | Step 2 |  |  | 447 |  |  |
|  | Step 3 |  |  | 1420 |  |  |
|  | Step 4 |  |  | 1210 |  |  |
|  | Step 5 |  |  | 774 |  |  |
| Other source |  |  |  | 4 |  |  |

Step 1: ‘Mechanomyography’.

Step 2: ‘Step 1’ AND (‘Systematic review’) AND ‘Review’.

Step 3: ‘Step 1’ AND ‘Muscle-function’.

Step 4: ‘Step 3’ AND ‘Muscle-assessment’.

Step 5: ‘Step 4’ NOT ‘Electromyography’.
