# Supplementary material for: Mechanomyogram for Muscle Function Assessment: A Review
Source: PLoS One. 2013 Mar 11;8(3):e58902. doi: 10.1371/journal.pone.0058902 (PMC3594217; doi:10.1371/journal.pone.0058902)
Supplement: Table S2 — Data extraction form for MMG in assessing muscle function. (DOC) [file pone.0058902.s002.doc]

**Table S2.** Data extraction form for MMG in assessing muscle function.

| **S/N** | **Details** | **Study** | **Subjects** | **Muscle/ Contraction** | **Sensor (Model)** | **Spectrum** | **With EMG** | **Parameters** | **Results** | **Application** | **Hardware /software** | **Signal Processing/Statistical analysis** | **Author’s Conclusion** | **Suggested Future Work** |
| --- | --- | --- | --- | --- | --- | --- | --- | --- | --- | --- | --- | --- | --- | --- |
| 1 | Kawakami et al., “Mechanomyographic activity in the human lateral pterygoid muscle during mandibular movement”, Journal of Neuroscience Methods 203 157– 162. **2012**. | Movement activity | Three male subjects without signs or symptoms of temporomandibular  disorders (age:  29.3  ±  2.5) | Lateral pterygoid (Jaw)/ maximal voluntary  clenching task | Condenser MIC/ B6P4FF05B (20Hz-20KHz, 2.5mm diameter, wt 2g, sensitivity 120 ±3dB) | (15-20)Hz | Yes (bipolar electrodes (Ag/AgCl)) | EMG amplitude Vs MMG amplitude | Correlated between MMG and EMG amplitudes for 20mm,  30mm jaw movements but not 10mm. | Not mentioned (NM) | SPSS 18.0(IBM Japan ltd), computer (real time), digital data recorder (PCM-D50), Magneton Vision MRI scanner, 3D motion capture system | FFT Hamming window, Pearson’s Correlation coefficient | The activity of the Lateral Pterygoid muscle could be evaluated by the MMG signals recorded in the external ear canal, unless the major jaw closing muscles show active contraction | Not suggests (NS) |
| 2 | W. Jeffrey Armstrong, “Wavelet-based intensity analysis of mechanomyographic signals during single-legged stance following fatigue”, Journal *of Electromyography and Kinesiology* 21 803–810, **2011**. | Postural control and fatigue study | 10 subjects (gender balanced, age: 25 ± 3 years). | Vastus lateralis, Soleus and Vastus medialis/NM | ACC (ADXL330, Analog Devices, Inc, Norwood, MA) | (5-100) Hz | No | Intensity (I) Vs Time, I Vs Wavelet index (j) , frequency cy Vs power | Peak MMG intensity was at lower frequency 12 Hz (j=3 ) for male and valley I was at higher frequency 42 Hz (j=6) for female, I increased with fatigue | Intensity analysis is useful for posture control and study the fatigue | PC, AcqKnowledge 4.0 (Biopac Systems, Inc), BPF (Blackman, 5-100 Hz), Data Acqusition unit (USB6008, National Instruments Austin, TX), PASW V 17.0 (SPSS Inc.,), LabVIEW Signal Express v3.0 (Austin TX) | Intensity analysis using wavelet, RM-ANOVA test | Analyzing MMG signals during single-legged stances using the Morlet wavelet intensity analysis provides insight into postural control strategy | Piper rhythm, changes in constraints affecting postural control, and changes in MMG (and EMG) intensity are warranted |
| 3 | Malek and Coburn, “Mechanomyographic Responses are not Influenced by the Innervation Zone for the Vastus Medialis”, Muscle Nerve 44: 424–431, **2011** | CE exercise effects on innervation zone | 10 healthy, men (age:24.4 ± 1.3 years) | Vastus medialis/ Cycle ergometry/ MMG response for IZ muscle action assessment | 3 ACC (EGAS- FT-10-V05, Entran) | 5-100 Hz | No | Norm. output power Vs absolute and norm MMG amplitude and MPF | MMG amplitude was no effect but was changed of MPF for each subject and sensor on distal, IZ and proximal to the muscle. | Can be used in monitoring muscular fatigue | Zero phase Butterworth BPF, ergometer, polar Heart Watch System, LabVIEW 7.1, SPSS 17.0, | DFT and Hamming Window for MPF, polynomial regression | The innervation Zone (IZ) does not influence the MMG signal during dynamic exercise | Vastus medialis may be used in future studies of muscular fatigue without regard for signal contamination by the IZ |
| 4 | Esposito et al, “Time course of stretching-induced changes in mechanomyogram and force characteristics”, *Journal of Electromyography and Kinesiology* 21 (**2011**) 795–802 | Stretching effect | 11 healthy males (age 22 ± 1 years) | Medial Gastrocnemious/ Isometric | Uniaxial ACC (ADXL202JE, Analog Devices, USA), | 4-120 Hz | Yes (silver/silver-chloride bars electrodes(diameter 1 mm, length 5 mm, inter-electrode distance 10 mm) for differential EMG detection) | Time Vs Rms , MF, for MMG, Time Vs rms , MF, CV for EMG and Time Vs pF | After stretching no significant different found by EMG, p-p and slope decreased -16% and -10% for MMG respectively, pF with 2 derivative decreased 35% | Can be useful for athletes | 12 bit data acquisition board, calibrated load cell (Mod. SM-200 N, Interface UK), PC, LiSin, Turin, Italy, SigmaStatv 3.11(Systat Software Inc, USA) EMGACQ | Peak-to-peak, time-to-peak, peak slope , ANOVA | Stretching altered significantly MMG and force signals. Also MMG RMS to pre-stretching values suggests that changes in viscoelastic parallel components recovered after few minutes. | No information exist on fluid behavior after stretching, thus further studies are required to gain more insights on this phenomenon. |
| 5 | Tanaka et al., "Study on evaluation of muscle conditions using a mechanomyogram sensor," *Systems, Man, and Cybernetics (SMC), 2011 IEEE International Conference on*, vol., no., pp.741-745, 9-12 Oct. **2011**. | Fatigue | Two healthy | Biceps brachii/Eccentric/muscle injury & triceps brachii/Isometric | Developed Piezoelectric based sensor | (5-100) Hz | No | Mean peak frequency (MPF) & variance Vs time | Rate of increase of variance with time declined & peak of MPF with time reached quickly for fatigue subject, | Monitoring muscle injury | Oscilloscope (Yokogawa Electric Corporation, DL1740), myodynamometer (ANIMA, µTas MT-1) | MPF and PSD by digital Fourier Transform | MMG sensor system for monitoring muscle conditions was developed, Muscle fatigue evaluation parameters of MPF and variance were suggested, | NS |
| 6 | Krueger et al, “Correlation between Mechanomyography Features and Passive Movements in Healthy and Paraplegic Subjects”, 33rd Annual International Conference of the IEEE EMBS Boston, Massachusetts USA, August 30 - September 3, **2011** | Knee angular movement | 12 healthy (age :31.45±4.56) and 13 spinal code injured (SCI) (age: 32.06±9.46) | Rectus femoris and vastus lateralis/knee extension | Freescale MMA7260Q MEMS triaxial ACCs with sensitivity equal to 800 mV/V at 1.5 G | 4-40 Hz | No | RMS integral, MF and skewness of MMG signal and knee angle | The correlation between MMG (MF) and MMG (RMS and integral) to healthy subjects was classified as positive, moderate (from 0.635 to 0.681) and high (from 0.859 to 0.870), and weak (positive e negative) to spinal code injured subjects | These results differ from those obtained in voluntary contraction or artificially evoked by functional electrical stimulation and may be relevant in applications with closed loop control systems. | Electrogoniometer, DT300 series Data Translation™, A LabVIEW™ program | Spearman correlation coefficients, Wilcoxon Signed Ranks Test | Both MMGμ3 and MMGMF are spectral analysis features and they showed antagonist responses to knee angle during passive movements. | Not reported |
| 7 | Natasha Alves and Tom Chau, “The design and testing of a novel  mechanomyogram-driven switch controlled by  small eyebrow movements”, *Journal of NeuroEngineering and Rehabilitation* **7**:22, **2010**, | Movement activities to control binary switch | 10 healthy individuals (5 Male; age 27 ± 2 years) | Frontalis/ eyebrow movements | Coupled MIC and ACC | 5-100 Hz | No | Time vs. RMS value of MMG, and frequency vs. CWT for 4 eyebrow movements | The switch showed almost perfect sensitivity and specificity for all participants. average sensitivity and specificity of the switch was 99.7 ± 0.4% and 99.9 ± 0.1%, | NM | 1KHz sampler (NI USB-6210), opto-isolator (4N36, Motorola Inc), LabVIEW, Visual Basic, | continuous wavelet transform (CWT) algorithm for contraction and baseline detection | the frontalis muscle is a suitable site for controlling the MMG-driven switch | Further investigation of the potential benefits of MMG-control for the target population is warranted |
| 8 | Xie et al., “ Uncovering chaotic structure in mechanomyography signals of fatigue biceps brachii muscle”, *Journal of Biomechanics* 43 1224–1226, **2010**. | Fatigue | Five healthy human subjects | Biceps brachii/Isometric contraction | ACC (EGAS-FS-19-V05, Entran Inc, Fairfield, NJ) | (5-250) Hz | No | Embedded dimension (*m)* Vs Correlation dimension ( *D_2_)* to study fatigue from nonlinearity | *D_2_* increased with *m* initially then entered into flat area at slight fluctuation | Rehabilitation, to prevent disorder, diagnosis fatigue | Cybex machine (Cybex Norm Testing and Rehabiliation System, Cybex Norm Int. Inc, USA), Adhesive tape , Matlab 7.0 | Volterra–Wiener–Korenberg (VWK) model approach for nonlinear detection,numerical titration method for Chaos detection | MMG is a high-dimensional chaotic signal and support the use of the theory of nonlinear dynamics for analysis and modeling of fatigue MMG signals. | Combining the surrogate data method with chaotic invariants may be potentially applied to differentiate the muscle states |
| 9 | Armstrong et al., “Reliability of mechanomyography and triaxial accelerometry in the assessment of balance”, Journal of Electromyography and Kinesiology 20 726–731, **2010**. | Balance | Five males and five females (mean age = 25 ± 3 yr) | Vastus lateralis, vastus medials & soleous/ NM | 3 ACC (ADXL330, Analog Devices, Inc., Norwood, MA), a wireless HRA ACC (G-Link, ±10g, Microstrain, Inc., Williston, VT) | 5-100 Hz | No | Trial Vs p-p acceleration of VT, ML and AP, Trial Vs ACC amplitude of VL,VM and SOL | Except RES but all measures demonstrated moderate-to-strong reliability (ICC=.75, .73, .63, .87, .89, .86 for VM,VL, SOL, VT,ML,AP respectively) | Can be used in clinical studies where forceplates are not available | Data acquisition unit (USB6008, National Instruments, Austin, TX), PASW v 17.0 (SPSS) for, LabVIEW Signal Express v 3.0, AcqKnowledge 4.0 (Biopac Systems, Inc., Santa Barbara, CA) | NM/ANOVA, ICC and Pearson’s correlation coefficient | MMG provide reliable information pertaining to balance, and may have application in evaluating postural control and stability. | relationships and predictability of these measures in controlled quasi-static positioning, more dynamic motions, and fatigue states |
| 10 | Hendrix et al., "Comparing electromyographic and mechanomyographic frequency-based fatigue thresholds to critical torque during isometric forearm flexion." Journal of Neuroscience Methods 194(1): 64-72, **2010**. | Fatigue threshold | 10 adults (4 men and 6 women, mean age = 22.0±2.1 years) | Biceps brachii/ Isometric | ACC (Entran EGAS FT 10, bandwidth 0–200 Hz, dimensions:1.0cm×1.0cm×0.5 cm,mass 1.0 g, sensitivity 10 mV/g) | 5–100 Hz for MMG and 10-500Hz for EMG | Yes , ( A bipolar surface (3.0cm center-to-center) electrode (circular 4mm diameter silver/silver chloride, BIOPAC Systems, Inc., Santa Barbara, CA, bandwidth 10.0–500 Hz). | MPF of MMG and EMG, and critical torque (CT) | There were no significant differences between fatigue thresholds (CT = 26.3± 0.8, EMG MPF_FT_ = 31.4±4.2, and MMG MPF_FT_ = 5±7.0%MVIC), and the mean torque values (Nm) from the three fatigue thresholds were significantly inter-correlated at r = 0.94–0.96. | May be used to examine the global motor unit firing rate of the unfused, activated motor units | Cybex II isokinetic dynamometer, a differential amplifier (Biopac Systems Inc., Santa Barabara, CA, bandwidth 10.0–500 Hz), LabVIEW programming software (version 7.1, National Instruments, Austin, TX) | NM/Linear regression, Pearson correlation, Statistical Package for the Social Sciences software (v. 17.0, SPSS Inc., Chicago, IL) | The EMG MPFFT test may provide a non-invasive method to examine the effects of interventions on the conduction velocity and shape of the action potential waveform. Activated motor units may be examined by the non-invasive methods of the MMG MPFFT test. | Future studies should examine EMG and MMG MPF responses during continuous muscle actions at the EMG MPF_FT_ and MMG MPF_FT_ to directly validate these tests. |
| 11 | Hendrix et al., "A mechanomyographic frequency-based fatigue threshold test." Journal of Neuroscience Methods 187(1): 1-7, **2010**. | Fatigue threshold | 9 adults (4 men and 5 women; age = 21.6±1.2 years) | Vastus lateralis, vastus medialis and rectus femoris/Isometric | Three ACCs (Entran EGAS FT 10, bandwidth 0–200 Hz, dimensions: 1.0×1.0×0.5 cm,mass 1.0 g, sensitivity 10 mV/g) | 5-100Hz | No | MMG MPF and torque | The isometric torque levels associated with the MMG MPF_FT_ for the three superficial muscles of the quadriceps. muscles | Non-invasive method to examine the effects of interventions such as caffeine, strength training, stretching, and fatigue of the muscles | Cybex II isokinetic dynamometer, LabVIEW programming software (version 7.1, National Instruments, Austin, TX), | Hamming window andthe discrete Fourier transform (DFT) algorithm/ Pearson correlation | The MMG MPFFT test may provide a non-invasive method to examine the effects of various interventions on the global motor unit firing rate during isometric muscle actions. | Future studies should compare the effects of continuous isometric, intermittent isometric and dynamic muscle actions on differences in the MMG MPF_FT_ of the VL, VM, and RF muscles |
| 12 | Taylor et al., Classifying human motion quality for knee osteoarthritis using accelerometers. Engineering in Medicine and Biology Society (EMBC), 2010 Annual International Conference of the IEEE, **2010**. | Exercise label of knee osteoarthritis | 9 (four males and five females, varying in height and weight). | Thigh & shin/ NM | SMB380 MEMS tri axial ACC (22grams, ±2g) | 0-25 Hz | No | Sample Vs acceleration | Assess exercise label of in correctness | At-home & clinic rehabilitation device |  | MATLAB for signal processing, WEKA software for data classification and analysis | The system will provide feedback on exercise performance based on the classifier decisions, motivate the patient to continue exercise, and report patient progress back to a physician and/or care giver. | In our next study, we will use patients who are currently undergoing physical therapy to verify that their errors are similar to these performed by our healthy subjects. |
|  |  |  |  |  |  |  |  |  |  |  |  |  |  |  |
| 13 | Scheeren et al, “Investigation of Muscle Behavior During Different Functional Electrical Stimulation Profiles Using Mechanomyography”, 32nd Annual International Conference of the IEEE EMBS Buenos Aires, Argentina, August 31 - September 4, **2010** | Muscle movement | 10 healthy (age=28.3±6.6 years) and 3 spinal cord injured (age=34.4±9.8 years) males | Rectus femoris and vastus lateralis/functional electrical stimulation (FES) | Freescale MMA7260Q triaxial ACC (800 mV/V at 1.5 g) | 4-40 Hz | No | RMS and MF of MMG | The lowest values for MMG RMS and MF parameters were verified in the 200-50 FES profile suggesting less muscle modification during the experiment. The MMG signal was different between healthy and SCI but there was no difference between the RF and VL muscles. | This study may be helpful creating experimental setups with FES walking performances and artificial functional movements control strategies. | A LabVIEW™ program, Data Translation™ DT300 series, Electrogoniometer | ANOVA test, least square difference *post hoc* test. | Using MMG technique and electrogoniometry simultaneously contribute to a better understanding of the muscle response to FES. |  |
| 14 | Tian et al., “Mechanomyography is more sensitive than EMG in detecting age-related sarcopenia”, Journal of Biomechanics 43, 551–556 **2010**. | Movement activity for age-related sarcopenia | 10 healthy elderly(64.574.5 yr) and 10 young(22.672.8 yr) | Vastus lateralis/isometric contraction | A biaxial ACC (weight 2gm, size: 5mmX5mmX8mm, measurementrange72g (g=9.81m/s2), and bandwidth DC—1000Hz.) | 5-100 Hz | Yes, EMG electrodes (Biovision, Wehrheim, Germany) (bandwidth=10–700 Hz) | RMS and MF of both MMG and EMG, and movement intensity | The MMG RMS differences between the young and the elderly across all three intensity level where EMG RMS was only different at the greatest intensity. | MMG could be used as an important measurement in studying muscle contraction in age-related sarcopenia. | DAQ unit DasyLab (version 6.0) software (DATALOG GmbH, Moenchengladbach, Germany), a leg extension machine (Cybex, Medway, MA, USA), Statistical Package for Social Sciences (SPSS) software program, version 10.0 (SPSS, Inc., Chicago, IL, USA). | Two-way ANOVA, a fast Fourier transformation (FFT) algorithm | Although all four main parameters, EMG RMS, MMG RMS, EMG MF and MMG MF, were different with differing movement intensities and group demographics, MMG was a more sensitive measure. |  |
| 15 | Herda et al., 2010, “A noninvasive, log-transform method for ﬁber type discrimination using mechanomyography”,Journal of Electromyography and Kinesiology 20 787–794, **2010** | ﬁber type discrimination | Five resistance-trained (RT) (mean ± SD age = 23 ± 3 years) 5 aerobically-trained (AT) (32 ± 5 years) and 5 sedentary (SED) (23 ± 4 years) men | Vastus lateralis/ isometric | An active miniature ACC (EGAS-FS-10-/V05, Entran Inc., Fairﬁeld, NJ) | 0-200 Hz | Yes (a bipolar surface electrode (20 mm center-to-center interelectrode distance; circular 4 mm diameter silver/silver chloride; Biopac Systems, Inc., Santa Barbara, CA)) | RMS of MMG and EMG, force and log terms | The AT group had the highest percentage of type I ﬁber area, the RT group had the highest percentage of type IIa ﬁber area, and the SED group had the highest percentage of type IIx ﬁber area. The lower b coefﬁcients for the AT group in the MMG RMS patterns may have reflected ﬁber area-related differences in motor unit activation strategies. | The present findings suggested that the information provided by both the MMGRMS and EMGRMS vs. force relationships is unique, yet this information could be used synergistically to interpret and for monitoring and describing the relationships. | Lab VIEW 7.1 software (National Instruments, Austin, TX),SPSS v. 12.0 (SPSS Inc., Chicago, IL)., performed isometric muscle actions of (York Barbell Company, York, PA),The biopsy sample was taken with U.C.H. needles (Popper and Sons, New Hyde Park, NY) using the double-chop method | ANOVA test | There are differences in ﬁber type composition of the vastus lateralis muscle among aerobically-trained, resistance-trained, and sedentary individuals | Not reported |
| 16 | Malek et al., “Comparison of Mechanomyographic Sensors During Incremental Cycle Ergometry for the Quadriceps Femoris”, Muscle Nerve 42: 394–400, **2010** | CE effect on MMG sensors | Nine healthy, college-aged men ( age 23.6 ± 0.8 years; | Vastus lateralis and rectus femoris/CE | ACC (Model EGAS-FT-10-/V05; Entran), PIZ sensor (Model 21050A; Hewlett-Packard, Andover, Massachusettts) | 5-100 Hz | No | Output power Vs MMG amplitude and MMG MPF | Polynomial regression analyses on a subject-by-subject basis indicated that the relationship between the normalized MMG amplitude versus normalized power output was best fit with either a linear, quadratic, or cubic model. These patterns were consistent between sensors for each muscle for each subject. No consistent relationship was found for MMG MPF within subjects and between muscle groups. | NM | A data acquisition system (MP 100WSW; Biopac Systems, Inc., Santa Barbara, California), LabVIEW 7.1, SPSS 16.0 | DFT & Hamming Window for MPF analysis, ANOVA & Polynomial regression analysis | For CE, both sensors provide similar information for the interpretation of motor control strategies during continuous exercise | NS |
| 17 | Scheeren et al., “Wrist Movement Characterization by Mechanomyography Technique”, Journal of Medical and Biological Engineering, 30(6): 373-380, Sep **2010** | Wrist movement | Twelve male healthy volunteers (24 ± 5.5 years) | Forearm/concentric/flexion, extension, radial deviation & ulnar deviation | ACC (MMA7260Q traxial, 800mV/V, 1.5 Gravitational acceleration) | (4-40) Hz | No | RMS, peak counting, zero crossing for four movement intensities | ANOVA test showed that both flexions and deviations were different from ulnar and radial, the module presented strong correlation between 0.2AOC (after onset of contraction) and 1.0AOC for both AWLs. | Can be used as motor prosthetic control | BPF, Data Translator ^TM^ (DT300), PASW Statistics^TM^ for Windows v.18, LabVIEW program | Zero-crossing and peak detection, t-test and Pearson’s correlation coefficient to verify difference , | The ability to identify distinct movements using two or more MMG sensors brings good perspectives to the development of new control strategy algorithms for driving upper-limb prostheses. | Studies larger number of limb movements to control strategy, such as pronation and supination of the forearm, or discrimination of fine movements or each finger individually. |
| 18 | Yoshimi et al., “Identification of the occurrence and pattern of masseter muscle activities during sleep using EMG and accelerometer systems”, *Head & Face Medicine*, 5:7, **2009**. | Mandible movement activities during Sleep bruxism |  | Masseter muscle/ clenching, grinding &tapping | 2 axis ACC ( ADXL 202E, Analog Devices Co. Ltd,Norwood, MA, USA), EMG (EMG, SN 700, Techno Science Co. Ltd, Tokyo, Japan ) | NM |  | Amplitude of clenching, grinding & tapping, Massester muscle activity Vs Bruxism length | Tapping was a rhythmic muscle activity with Y-axis movement, clenching was strong muscle activity with no Y-axis movement, and grinding was muscle activity with X and Y movement. | NM | EEG (Poly mate AP1124, TEAC Co. Ltd, Tokyo, Japan), Infrared video camera, Laser Doppler Flowmetry (CDF-2000, Cyber Med, OAS Co., Japan), SPSS 13.0 (ANOVA), Bruxism Analysis Software (G1 System Co. Ltd Tokyo, Japan) | NM/ANOVA and Tukey HSD test | the tapping, clenching, and grinding movement of the mandible could be effectively differentiated by the new system and sleep bruxism was predominantly perceived as clenching and grinding, which varied between individuals | NS |
| 19 | Faller et al., “Muscle fatigue assessment by mechanomyography during application of NMES protocol”, Rev Bras Fisioter, São Carlos, v. 13, n. 5, p. 422-9, Sept./Oct. **2009**. | Fatigue | 10 healthy males (age= 26.7±5.35 years) | Rectus femoris/ Isometric | Triaxial ACC (as reference 25,26) | 4-40 Hz | No | Time Vs normalized torque, rms and MPF of MMG signal | MMG rms correlated with torque but MMGmpf did not correlated significantly with torque at present NMES | To assess functional movement for NMESed muscle contraction | Butterworth BPF, 12-bit ADC, signal generator (PASCO Digital Function, PI-9587), LabVIEW (NI, Austin, TX), , | FDFT algorithm and Hamming Window to obtain PSD, cross correlation | MMG is a technique that can be simultaneously applied to NMES because there is no electrical interference and it can be used during functional movements in the NMES-generated muscle contraction. | NS |
| 20 | Al-Zahrani et al., “Within-day and between-days reliability of quadriceps isometric muscle fatigue using mechanomyography on healthy subjects”, Journal of Electromyography and Kinesiology 19 695–703, **2009**. | Fatigue reliability within day and between days | 31 healthy subjects (15 males) | Rectus femoris/Isometric | Triaxial ACC (ENDEVCO Model 7253C-10, Germany; 3.6g, sen 10mV per unit gravitational acceleration) | 5-100 Hz | No | Time Vs rms amplitude, MPF, MF/ ICC to assess reliability | Low reliability and large error for between days of MPF and MF respectively, overall, ICC were high reliable for MPF and lower SDD for MF | NM | MVC measurement dynamometer (ISOCOM, Isokinetic technology, Nottingham, UK), USB data acquisition card (NI, USA), 3 channel charge amplifier (ENDEVCO Inc, Germany), LabVIEW 8.0 (NI, Austin, TX), SPSS 14.0 | FIR filter to exclude low frequency vibration, ICC, SEM, SDD | Results of the current study show that MMG RMS, MPF and MF linear regression slopes from rectus femoris muscle are not suitable for the monitoring of muscle fatigue due to the high SDD values | NS |
| 21 | Xie et al., Detection of chaos in human fatigue mechanomyogarphy signals. Engineering in Medicine and Biology Society, 2009. EMBC 2009. Annual International Conference of the IEEE, **2009**. | Fatigue signal nature | 5 subjects | Biceps brachii/Isometric | ACC (EGAS-FS-10-V05, Entran Inc, NJ) | 5-250Hz | No | Linearity and nonlinearity of fatigue during contraction | MMG signals in fatigue state of all observed subjects were a chaotic signal, and were generated by nonlinear dynamics systems | For the analysis and modeling of the MMG | NM | Volterra-Wiener-Korenberg model to detect nonlinearity, Gaussian kernel algorithm to determine the correlation dimention | MMG is a high-dimensional chaotic signal and support the use of the theory of nonlinear dynamics for the analysis and modeling the MMG signals | NS |
| 22 | Feng, et al., Mechanomyogram for identifying muscle activity and fatigue. Engineering in Medicine and Biology Society, 2009. EMBC 2009. Annual International Conference of the IEEE, **2009**. | Fatigue | Five healthy subjects, ages ranging from 21 to 32 years (four males and one female). | Biceps brachii/Isometric | Electret Condenser MIC (MX183, Shure Cardioid Condenser Lavalier) | 0-500Hz | No | %MVC Vs RMS and MF | RMS increased with increase in the force of contraction, there is significant change in the RMS with the onset of fatigue, consistent decrease in the value of MMG with muscle fatigue. | NM | 32 bit ADC, Adobe audio software for segmenting, MATLAB 2008b | RMS and MF /Mean and SD | There is a consistent decrease in the RMS value of MMG with muscle fatigue but MF of the MMG was not a measure of the strength of contraction or muscle fatigue and varied erratically | would improve the understanding of the size and location of microphone, and determine the impact of gel applied to the surface of the microphone prior to determining the efficacy of MMG to identify muscle activity |
| 23 | Malek, et al., “Comparison of mechanomyographic amplitude and mean power frequency for the rectus femoris muscle: Cycle versus knee-extensor ergometry”, *Journal of Neuroscience Methods* 181 (**2009**) 89–94 | Muscle action during knee extensor and cycle ergometry (CE)/ | Eight healthy men (age: 27.3±2.3 years) | Rectus femoris/ Knee extension (KE) | ACC (Entran, EGAS-FT-10-/V05) | 5-100 Hz | No | Norm. output power Vs absolute and normalized MMG amplitude and MPF | Knee extensor resulted in similar patterns of responses for MMG amplitude for the composite data and all 8 subjects, but MPF was inconsistent | Suggest to use KE for dynsmic action & CE for fatigue during cycling | Zero phase Butterworth BPF, Data acquisition unit (MP 100, BIOPAC System, Inc,Santa Barbara CA), PC, ergometer (Calibrated Quinton Corval 400), LabVIEW 7.1, SPSS 15.0, | DFT and Hamming Window for MPF, polynomial regression, t-test and F-test | Knee-Extensor rather than traditional Cycle E rgonometry exercise may be an optimal mode of examining MMG amplitude for the RF muscle | The motor control strategies of the quadriceps muscles for dynamic exercise should use the KE model & CE model should be used to examine neuromuscular fatigue |
| 24 | Ebersole et al., “Fatigue and the Electromechanical Efficiency of the Vastus Medialis and Vastus Lateralis Muscles*”, J Athl Train*. Mar-Apr; 43(2): 152–156, **2008**. | Fatigue | 10 healthy males (age = 23.2 ± 1.2 years) | Vastus medialis & Vastus lateralis/concentric isokinetic leg extension | PIZ (Model 21050A; Philips Medical Systems, Bothell, WA; 0.02-2000Hz), Bipolar Surface electrodes (model MeshTrode) | 5-100 Hz for MMG, 10-500 Hz for EMG | Yes, bipolar surface electrodes (model MeshTrode [rectangular solid gel, silver-silver chloride snap connector]; Verimed International Inc, Coral Springs, FL) | Torque, electromechanical efficiency (EME), slope | Linear regression confirmed the decrease in torque (0.96), EME for VM (0.73) and VL (0.73), slopes were same for VM and VL EMEs | Assessing and quantifying knee injury at clinically | Biodex System 3 Dynamometer, Shirley NY, Singal interface Unit (model DI-220), LabVIEW 7.0, WinDaq Software | RMS MMG and Peak torque as signal processing by LabVIEW, Polynomial regression analysis by SPSS 11.5 | EME may be sensitive to distinguish healthy and injured muscle having atrophy or dysfunction but knee joint disorders | Future researchers should examine EME from these muscles in a clinical population as well as in response to specific interventions |
| 25 | Krizˇaj, et al., “Short-term repeatability of parameters extracted from radial displacement of muscle belly*”, Journal of Electromyography and Kinesiology* 18 645–651, **2008**. | Fatigue rate | 13 healthy males (age= from 19 to 42 years) | Bicep Brachii/NM | A digital displacement sensor, DDS (G40, RLS Inc) | NM | No | Time Vs muscle belly Max displacement, delay, contraction, sustain and half relaxation times | For all parameters ICC were above 0.86 meant good short-term repeatability, Normalized standard error was lower than 2% meant high precision | NM | Linear steeping motor controlled by a PC, | Intra-correlation coefficient (ICC) to measure repeatability, Normalized standard error mean (NSEM) to measure reliability | Maximal displacement and half relaxation time show largest influence to muscle fatigue rate and are also expected to be the best measure of the fatigue rate. | Further studies of long-term repeatability should be performed. |
| 26 | Ryan et al., “Inter-individual variability in the torque-related patterns of responses for mechanomyographic amplitude and mean power frequency”, *Journal of Neuroscience Methods* 161 212–219, **2007**. | Strength | Twelve healthy men (age = 25±4 years). | Vastus lateralis/Isometric | Miniature ACC (EGAS FS-10-/VO5, Measurement Specialities Inc., Hampton, VA) | 5-100 Hz | No | Time Vs torque/ MMG_RMS_, Isometric % MVC Vs MMG_RMS_ and MMG_MPF_ | MMG amplitude versus isometric torque relationship was best fit with a linear model for the LS group and a cubic model for the HS group, MMG MPF was best at linear for both the group, | NM | Biodex Systems 3 dynamometer,cycle ergometry, data acquisition unit MP150WSW, Biopac System | labVIEW 7.1, Butterworth LPF, Hamming Window, DFT, Polynominal regression, SPSS 12.0 | strength differences do not affect the patterns of responses for MMG amplitude or MPF | Future studies should examine the individual patterns of response to draw conclusions about motor control strategies. |
| 27 | Cramer et al., “Acute effects of static stretching on characteristics of the isokinetic angle – torque relationship, surface electromyography, and mechanomyography”, *Journal of Sports Sciences, April* **2007**; 25(6): 687 – 698 | Stretching effect on muscle strength | 10 women (age 23.0+2.9 years, and 8 men (age 21.4+3.0 years) | Rectus femoris/concentric and isokinetic | Miniature ACC (EGAS-FS, Entran, Inc., Fairfield, NJ), Bipolar Ag-AgCl (Moore Medical), calibrated Biodex 3 Dynamometer (Biodex Medical Systems, Inc., NY) | 5-100, 10-500 Hz for MMG & EMG respectively | Yes (Bipolar surface electrode (Moore Medical, Ag -AgCl)) | Joint angle Vs Peak torque (pT), Acceleration time, EMG and MMG amplitudes | PT, acceleration time, and EMG amp decreased from pre- to post-stretching at 1.04 and 5.23 rad /s; no changes in work, joint angle at PT, isokinetic range of motion, or MMG amp . | Sports application: Can guide to the athletes | PT measurement by TAE (torque acceleration energy) | Butterworth BPF, AcqKnowledge III software for EMG & MMG rms values, SPSS v 11.5 for lower order ANOVA test | Static stretching appears to affect muscle strength at slow and fast speeds, and thus may affect all types of athletes | The volume of stretching necessary to safely increase joint range of motion before performance, but not elicit detrimental changes in muscle force production that could adversely affect performance |
| 28 | McKay et al., “Resting mechanomyography before and after resistance exercise”, Eur J Appl Physiol 102:107–117, **2007** | Exercise effect on muscle mechanical signal | 10 healthy, moderately fit young men age (23.0 ± 2.3 years) | Rectus femoris/ resistance exercise | ACC (Bruel & Kjaer #4381; 43 gm; 2X2 cm; Bru¨el & Kjær S & V, Denmark) | 0.2 to 100 Hz. | Yes, a commercially available Ag-Agcl electrode (Meditrace 200, The Ludlow Company LP, Chicopee, MA, USA) | RMS of MMG and EMG, normalized MMG amplitude over time | Resting MMG amplitudes increase about threefold after vigorous resistance exercise, and that the increase decays exponentially over time. Importantly, all subjects demonstrated an increase ranging from 1.8 to 7.7 times the pre-exercise level. Resting-muscle surface EMG amplitudes doubled after resistance exercise, but the amplitudes were below the resolution of the instrument. | The method and the phenomenon may have important implications in the study of metabolism, exercise, and muscle physiology. | SigmaStat for Windows V. 3.11, Jandell Corp, San Rafeal, CA, USA).SPSS V. 10 (SPSS Inc. Chicago, USA), Easyplot V. 4.0.4 (Spiral Software, Massachusetts Institute of Technology, Boston, MA, USA), MatlabTM (The MathWorks, Inc. Natick, MA, USA), and VMAX 29 Series metabolic cart for oxygen consumption mesurement (Sensormedics, Yorba Linda, CA, USA). | Standard Error of the Estimate, Gauss–Newton algorithm, Fast Fourier Transforms, Autocorrelation and ANOVA | Resting muscle is more mechanically active following resistance exercise and that this may contribute to elevated oxygen consumption. | To examine in future whether resting-muscle MMGs change with muscle disease or with alterations in muscle tone or atrophy |
| 29 | Ioi et al., “Mechanomyogram and electromyogram analyses for investigating human masseter muscle fatigue”, orthodo n t i c wa v e s 6 5 1 5 – 2 0, **2006**. | Fatigue | 16 healthy Japanese males (aged 25.6±2.3 years) | Masseter/ voluntary biting force | Amorphous sensor (30x9mm, weight 17g, resolution 0.02µm) | Set upper cutoff frequency at 300 Hz for MMG and 3000 Hz for EMG | Yes (EMG surface electrodes with 51mm inter-electrode distance) | %MVC Vs average rectified value (ARV) of MMG ,EMG and electromechanical efficiency | ARV for MMG raised up to 20% then started to fall, a nonlinear and linear relationship bet’n MVC & ARV for pre or post fatigue for MMG and EMG respectively, EME was lower at post fatigue | Useful for evaluating muscle status | A small rare-earth magnet (3.5x1mm, Wgt=0.06g),bite-force transducer (MPM-3000; Nihon Cohden Co., japan),PCM recorder | t-test to compare mean difference of ARV for MMG and EMG | These findings suggest that the MMG analysis combined with the EMG may be a more useful method for evaluating the masseter muscle status. | Additional investigation on the issue of the relationship between force and the MMG activity appears to be warranted |
| 30 | Gobbo et al., “Torque and surface mechanomyogram parallel reduction during fatiguing stimulation in human muscles*”, Eur J Appl Physiol* 97: 9–15, **2006**. | Fatigue | 10 healthy sedentary male subjects (age 20–50 years old) | BB & VL/Isometric | Uniaxial ACC (ADXL202JE, Analog Devices,Inc., USA) | 0-128Hz | No | Fatiguing cycle Vs norm MMG and torque/ Peak torque (PT) Vs MMG p-p for correlation | For both muscles % MMGp-p and %PT decreased more in VL, with increasing fatigue/ %PT and %MMGp-p had a high correlation for both BB & VL | Monitoring fatigue in sport training or rehabilitation protocol | Calibrated load cell (SM-100 N, operating range 0-100N), | Normalization and correlation of MMGp-p and PT, | surface MMG detection may find clear and useful practical applications for monitoring the mechanical fatigue growth, in order to avoid potential stress disorders | NS |
| 31 | Madeleine et al., “Spectral moments of mechanomyographic signals recorded with accelerometer and microphone during sustained fatiguing contractions”, *Med Biol Eng Comput* 44: 290–297, **2006**. | Fatigue | 14 healthy male volunteers (right-handed) (age= 26.7±4.9 years) | Biceps brachii/Isometric/ | Air coupled condenser MIC (BCM 9765, BeStar Acoustic, China, 9.7mm dia, 18g weight), pzo ACC (Bang & Olufsen Technology, Struer, Denmark, 17.6 dia, 2.9 g weight, sen:30pC/ms-2) | 1-500 Hz for MP, 1-100 Hz for ACC, 2-100 Hz for offline analysis | No | Frequency Vs MMG (ACC & MP), Ttime Vs rms, normalized, Coefficient of variance, Mc2 and µ3 of MP and ACC MMG signal | For both MMGMIC and MMGACC, absolute and normalised RMS and Mc2 increased while MNF and µ3 decreased with contraction time, The rates of change of RMS over time were significantly correlated for both but not correlated for spectral moments | NM | 14 bit/12 bit ADC,BPF,MMG amplifier (MP & ACC) , | Welch Periodgram with Hamming Window for PSD analysis, ANOVA, Skewness, CoV | Higher order spectral moments of the MMG signal change during sustained contraction, indicating a complex modification of the shape of the power spectrum and not just scaling of the bandwidth. | NS |
| 32 | McKay et al., “Effects of graded levels of exercise on ipsilateral and contralateral post-exercise resting rectus femoris mechanomyography”, *Eur J Appl Physiol* (**2006**) 98:566–574 | Muscle activity of exercise | 10 fairly healthy (6 males and 4 females) (age:33 ± 13 years) | Rectus femoris/concentric | ACC (Bruel and Kjaer, # 4381, Naerum, Denmark) | 2-100 Hz | No | Repetitions Vs work, correlation between work & normalized mean absolute acceleration, | MMG and work was Linearly correlated, non-exercise thigh was half in activity compare to exercise thigh, MMG activity was higher at shorter length of RF muscle | NM | A Biodex 3 dynamometer (Biodex Medical Systems Inc.,Shirly, USA),SigmaStat for Windows Version 1 (Jandel Scientific , USA) | Standard Error of measurement (SEM), ICC and regression for correlation measurement, ANOVA | EPERMA is correlated linearly with the increase in exercise work, muscle resting in its shortened position increases EPERMA, there is a cross-over effect of the increase in EPERMA to the corresponding contralateral non-exercised muscle | EPERMA is likely neurally mediated, although further evidence is needed |
| 33 | Matta et al., “Interpretation of the mechanisms related to the muscular strength gradation through accelerometry”, Rev Bras Med Esporte _ Vol. 11, No. 5, **2005** | Strength | 15 male (with ages 24.0 ± 5.25 years), and 12 female (ages 21.7 ± 1.5 years), | Brachii Biceps / Isometric | Biaxial ACC (ADXL 202E Analog Devices USA), band 200Hz, mass 1.5g, sens: 315mV/g, range upto 2g | NM | No | Male/ female Vs RMS and MF at 20 to 100 % maximum workload | RMS in X axis and Y axis increased with workload for both male and female, but MF for male was almost stable and slightly decreased for female with workload for both axes | NM | 12 bit ADC, A dynamometer (Kratos Dinamometeros), | LabVIEW 5.0, FFT for spectral analysis, Statistica Software 6.0 ANOVA (StarSoft, USA) | During the muscular contraction, there is non-uniform variations on the fiber’s diameter, besides the low frequency lateral oscillations | NS |
| 34 | Marek et al., “Acute Effects of Static and Proprioceptive Neuromuscular Facilitation Stretching on Muscle trength and Power Output”, *Journal of Athletic Training*;40(2):94–103, **2005** | Strength | 10 female (age, 23 6 3 years) and 9 male (age, 21 6 3 years) apparently healthy | VL & RF/Concentric isokinetic | Miniature ACC (EGAS-FS-10-/V05, Entran Inc., Fairfield NJ), sens:70mV/ms-2, range ±98ms-2, bandwidth: 0-200Hz | 10-500Hz for EMG & 5-100 Hz for MMG | Yes (Pregelled, disposable EMG electrodes containing a 1 cm diameter Ag-AgCl disc (Moore Medical, New Britain, CT)) | Peak torque (PT), mean output power (MP), active & passive range of motion (ROM), MMG, EMG amplitudes | PT, EMG, MP decreased for both static and PNF stretching at 60 & 300^o^/s, AROM & PROM increased for both stretching, MMG amplitude increased for RF muscle at 60^o^ static stretching but not change other cases | Can be useful to help clinicians for rehabilitation progress | Biodex System 3 dynamometer, Biopac data acquisition unit (MP150WSW), goniometer, EMG electrodes | LabVIEW 6.1 for signal duration for contraction, AcqKnowledge III for RMS values, SPSS 11.5 and Excel 2003 for mean, ANOVA, paired *t*-test | Both static and proprioceptive neuromuscular facilitation stretching caused similar deficits in strength, power output, and muscle activation at both slow (608·s21) and fast (3008·s21) velocities. | Further research is needed to examine the effect of pre-exercise stretching on muscle strengthening and/or strength assessments in athletes or patients who have experienced a muscle, tendon, or joint injury |
| 35 | Beck et al., “Comparison of Fourier and wavelet transform procedures for examining the mechanomyographic and electromyographic frequency domain responses during fatiguing isokinetic muscle actions of the biceps brachii”, *Journal of Electromyography and Kinesiology* 15 190–199, **2005**. | Fatigue | Seven men (age = 23 ± 3 years) | Biceps brachii/Isokinetic | PIZ (Hewlett-Packard, 21050A, bandwidth 0.02-2000Hz, Andover, MA), Bipolar electrode (Quinton Quick prep Ag-AgCl, Santa Barbara, CA) | 5-100Hz for MMG, 10-500Hz for EMG | Yes (Bipolar (7.62 cm center-to-center) electrode (Quinton Quick prep Ag–AgCl, Santa Barbara, CA)) | Repetition number Vs normalized frequency interms of MPF, MDF and CF of both MMG and EMG signals | Significant correlation between MPF,MDF,CF for both EMG and MMG, all these parameters decreased with increase of repetitions number | Can use to assess dynamic fatigue using motor unit strategy | Cybex II dynamometer, PC, LabVIEW 6.1, SPSS, FFT & CWT | LabView and FFT and/or CWT algorithms for Center frequency CF analysis, Polynomial regression for zero order correlation among normalized MPF,MDF and CF | Fourier based methods are acceptable for determining the patterns for normalized MMG and EMG center frequency during fatiguing dynamic muscle actions. | NS |
| 36 | B. Gregori, E. Galie and N. Accornero, “Surface electromyography and mechanomyography recording: a new differential composite probe”, *Med. Biol. Eng. Comput*., 41,665-669, **2003**. | Fatigue | Normal subjects | Biceps brachii/Isometric | Single probe combined with two piezoelectric ceramic discs (Stettner and Co TS-50-06-9 or similar) and EMG electrodes, size: 3x20x0.2mm, 1Hz-100KHz, wg:35g | 2Hz-2KHZ | Yes (EMG electrodes, 25mm inter-electrode distance) | Time Vs EMG and MMG amplitude, differential and non-differential MMG | Differential amplification significantly improved the signal-to-noise ratio in MMG recordings and significantly suppressed artifacts | useful in studying fatigue and neuromuscular diseases | A single sided PCB board, AD 524 differential amplifier, ADC (PICO technology) | Spectrum analysis/NM | The composite probe recorded muscular activity more efficiently than the non-differential probe and could therefore this method could provide useful information on muscle activity, even in a routine clinical settings | NS |
